# Polymorphism of rs12294045 in *EAAT2* gene is potentially associated with schizophrenia in Chinese Han population

**DOI:** 10.1186/s12888-022-03799-1

**Published:** 2022-03-08

**Authors:** Lina Wang, Tantan Ma, Dongdong Qiao, Kaiyan Cui, Xiaojiao Bi, Chao Han, Limin Yang, Mengmeng Sun, Lanfen Liu

**Affiliations:** Department of Psychiatry, Shandong Mental Health Center, Shandong University, No. 49 Wenhua Dong Road, Lixia District, Jinan, 250014 Shandong China

**Keywords:** *EAAT1*, *EAAT2*, Schizophrenia, Symptom, Cognitive deficits

## Abstract

**Background:**

Recent studies have shown that the excitatory amino acid transporters (EAATs) are associated with schizophrenia. The aim of this study was to investigate the relationship between the polymorphism of *EAAT1* and *EAAT2* genes and schizophrenia in Chinese Han population.

**Methods:**

A total of 233 patients with schizophrenia and 342 healthy controls were enrolled. Two SNPs in *EAAT1* gene (rs2269272, rs2731880) and four SNPs in *EAAT2* gene (rs12360706, rs3088168, rs12294045, rs10836387) were genotyped by SNaPshot. Clinical features were collected using a self-made questionnaire. Psychotic symptoms of patients were measured by the Positive and Negative Syndrome Scale (PANSS), and patients’ cognitive function was assessed by Matrics Consensus Cognitive Battery (MCCB).

**Results:**

Significant difference in allelic distributions between cases and controls was confirmed at locus rs12294045 (*Ρ* = 0.004) of *EAAT2* gene. Different genotypes of rs12294045 were associated with family history (*P* = 0.046), in which patients with CT genotype had higher proportion of family history of psychosis. The polymorphism of rs12294045 was related to working operational memory (LNS: *P* = 0.016) and verbal learning function (HVLT-R: *P* = 0.042) in patients in which CT genotype had lower scores. However, these differences were no longer significant after Bonferroni correction.

**Conclusions:**

Our study showed that the polymorphism of rs12294045 in *EAAT2* gene may be associated with schizophrenia in Chinese Han population. CT genotype may be one of the risk factors for family history and cognitive deficits of patients.

## Introduction

Schizophrenia is a common psychiatric disorder with unclear etiology characterized clinically by positive symptoms, negative symptoms, and cognitive deficits [1]. Cognitive deficits are now generally recognized as core symptoms of schizophrenia, which have their own pathologic mechanisms and related to the abnormal development of the nervous system [2].

The glutamate hypothesis of schizophrenia suggests that the abnormal transmission of glutamate in the brain leads to cognitive and behavioral abnormalities related to schizophrenia [3, 4]. The changes of glutamate concentration in the central nervous system may damage the structural connection and integrity of neurons and cause programmed cell death and cell proliferation. Such changes may affect the ability to adapt to environmental changes and resist various physiological injuries. Approximately 40% of the synapses in the central nervous system are glutamatergic synapses [5, 6]. When glutamate is at normal level, it plays an important role in synaptic plasticity, learning and memory, neurodevelopment and so on [6]. The increased level of glutamate in the glutamatergic synaptic gap could cause excessive activation of the corresponding glutamate receptors, resulting in excessive increase of intracellular calcium concentration in neurons, leading to excitatory neuron damage or even nerve cell death [7, 8]. Relevant studies have shown that this excitotoxicity may be associated with the occurrence and development of a variety of neuropsychiatric disorders including schizophrenia, bipolar disorder, attention deficit hyperactivity disorder, epilepsy, stroke, amyotrophic lateral sclerosis, and idiopathic tremor [9–12]. On the other hand, the N-methyl-D-aspartate (NMDA) hypofunction hypothesis of schizophrenia indicated that the inhibition of synaptic NMDA receptors which cause decreased glutamate release was related to schizophrenia [13, 14]. And the glutamate level in the synaptic space is mainly regulated by excitatory amino acid transporters (EAATs) [15–17].

Previous studies have found that *EAAT1* and *EAAT2* genes are associated with schizophrenia. Region-specific increases in cortical EAAT1 and EAAT2 mRNA were involved in schizophrenia pathophysiology [18, 19]. The expression levels of EAAT2 protein in patients’ brain were lower than those in normal [20–22], and the impaired cognitive functions such as speech fluency and verbal learning function in patients were related to the decreased expression level of this gene [15, 23]. In addition, *EAAT2* gene polymorphisms were found to be associated with schizophrenia in Japanese populatio [24], and the G allele of SNP rs4354668 was associated with poor clinical manifestations in abstract thinking and working memory [25, 26]. According to the glutamate hypothesis of schizophrenia [27–30], the researchers suggested that decreased levels of glutamate in schizophrenia may be associated with impaired cognitive and social functioning [31, 32].

Therefore, we conducted a case-control study to investigate the relationship between the polymorphisms of *EAAT1* and *EAAT2* genes and clinical characteristics, symptoms severity and cognitive deficits in Chinese Han patients with schizophrenia.

## Methods

### Participants

A total of 233 Patients, hospitalized in Shandong Mental Health Center of China from November 2015 to March 2018, who met the Diagnostic and Statistical Manual of Mental Disorders-Fourth Edition (DSM-IV) diagnosis of schizophrenia were enrolled in this study. Patients also met the following criteria: ages were range between 18 and 60 years old; biological parents were Chinese Han population; they were able to cooperate in completing the Matrics Consensus Cognitive Battery (MCCB) cognitive test; they have not been on antipsychotic medication for at least one month or receive modified electric convulsive therapy for six months; they had no history of drug or cigarette or alcohol abuse. Meanwhile, we included 342 healthy controls aged between 18 and 60 years old. They also met the criteria including their biological parents were Chinese Han population, they had no family history of mental illness, and they had no substance abuse and no pregnancy plan in the near future.

### Assessments

A self-made general clinical data questionnaire was used to collect the participants’ data including age, gender, education level, occupation, age of onset, disease duration, family history, interpersonal relationship, marital status and other disease-related data. The Positive and Negative Symptom Scale (PANSS) was used to assess the symptoms severity of the patients. The patients’ cognitive function was assessed using Matrics Consensus Cognitive Battery (MCCB) which has been shown to be an effective tool for testing cognitive deficits of Chinese patients with schizophrenia [33].

### SNPs selection

The SNPs information of *EAAT1* and *EAAT2* genes in Chinese Han population was downloaded from the International Genome Database and the Genome Reference Consortium Human Genome Build 38 (GRch38) edition and then were analyzed by Haploview software (version 4.2). Tag SNPs were screened according to MAF > 0.05 and Υ^2^ ≥ 0.8. Two loci of *EAAT1* (rs2269272 and rs2731880) and four loci of *EAAT2* (rs12360706, rs3088168, rs12294045 and rs10836387) were selected.

## DNA extraction and SNPs genotyping

A total of 5 ml of peripheral venous blood collected from patients and healthy controls was placed in anticoagulant tubes containing 0.5 mol/L ethylenediamine teacetic acid. The tubes were then centrifuged for 10 min at the speed of 3000 rpm/min to remove white blood cells and serum. The deoxyribonucleic acid (DNA) of the blood sample was extracted using the modified potassium iodide method.

The primers were designed using the online primer design tool and synthesized by The Beijing Genomics Institute (Beijing, China) (Table 1). The PCR was carried out using 29-μl volume including 2 μl genomic DNA, 3 μl of 10 Ex Taq buffer (Takara), 1 μl of MIX Primer, 2 μl of dNTPs (2.5 mM each), 20.8 μl of H_2_O and 0.2 μl of Ex Taq [5 U/μl] (Takara). The reaction conditions of PCR were as follows: denaturation at 96 °C for 20 s after an initial step of 2 min at 96 °C; annealing at 54 °C for 10 s; extending at 72 °C for 30 s; 35 cycles in total. Then the amplified products were digested and purified. The SNaPshot extension reactions were performed with the SNaPshot Multiplex PCR Kit (Applied Biosystems) using 5 μl volume including 3 μl PCR product, 1 μl of MIX Primer (5 PM), 0.5 μl of SNaPshot MIX (ABI) and 0.5 μl of ddH_2_O. The reaction process was as follows: denaturation at 95 °C for 10 s after an initial step of 2 min at 95 °C; annealing at 50 °C for 5 s; extended at 60 °C for 30 s; 35 cycles in total. After the above reaction, 5ul of SNaPshot PCR production with 0.5 U of SAP was performed at 37 °C for 60 min and 75 °C for 15 min. Finally, genetic analysis of 8 μl volume including 1 μl SNaPshot product, 6.5 μl of Hi-Di Formamide and 0.5 μl of GS-120 LIZ (ABI) was carried out using the PRISM 3730 XI Genetic Analyzer (Applied Biosystems), and the results were analyzed with GeneMapper v4.1 software (Applied Biosystems).

**Table 1:** List of primer pairs for multiple PCR

Gene	Primer	Sequence (5′–3′)	Length (bp)
EAAT1	rs2269272-F	TCCTTAGAATGAGGGAAAC	258
	rs2269272-R	CAGCGTCTTTGACTGGATA	
	rs2269272-YF	ATAAGAGAAATGGTAGAAGATGAATCAGTATGAAGACACTGT	42
	rs2731880-F	TTTGTAAATGCTCCTCCTGC	455
	rs2731880-R	CAAACATTGAGCAACCACTG	
	rs2731880-YF	TGACGGCCTACTGCCAACAGAAGGTTATGATACTGT	36
EAAT2	rs12360706-F	GGGAAGTAACTCTTATGGA	281
	rs12360706-R	AACTGACTGTTAGCCTTGT	
	rs12360706-YF	CCTCAGAGATGTGCTGGACCAACTTCCTTGGCTAGT	36
	rs3088168-F	TATAGATGCTCTGTGCTACGTGACT	282
	rs3088168-R	AAGGGTAAAGCCTACAATA	
	rs3088168-YF	TGAAAGGAGTTGAAGAAGCCACATTTTCAAGGAAAAATTAGCCTGTCCACCATA	54
	rs12294045-F	CATGCCCTCAAAGATCTAAGGTAAA	300
	rs12294045-R	CAGTTACAGCAGGCCAGAA	
	rs12294045-YF	ACTTGGGTTTCTCAAAGGGCAAGAATGAGAAAGAGAAGAATTAAAGTCTACTTAGTTGGTTTTCTC	66
	rs10836387-F	CTGCGTGAGTTGCTGATTC	246
	rs10836387-R	GTTGTCTTCTATTGCCTGA	
	rs10836387-YF	AATCTGTAGGGAGAAGCTGAGCTGCACTGGATGACTGTTATGCTCCCA	48

### Statistical analysis

Statistical analyses were carried out with the SPSS package (version 21.0). The goodness-of-fit Chi-square test was used to verify whether the allele and genotype frequencies of the members in case group and control group corresponded to the Hardy-Weinberg equilibrium (HWE). The chi-square test was used to analyze the differences between the various qualitative data of the subjects, including allele frequency and genotype frequency, gender, onset form, family history of psychosis, interpersonal relationship, premorbid characteristics, current marital status, occupational status, etc. One way analysis of variance (ANOVA) were used to compare the differences in age of onset, total disease duration, PANSS scores (except PANSS Positive scores) and cognitive function of patients with schizophrenia. PANSS Positive scores was carried out with Kruskal-Wallis test for non-parametric test. The Least Significant Difference (LSD) method was used to perform multiple analysis. Linkage disequilibrium analyses and haplotype analyses were performed by Haploview software (Version 4.2).

## Results

A total of 575 subjects were enrolled in this study in which 233 were patients with schizophrenia and 342 were healthy controls. There was no significant difference in gender (*χ2* = 0.041, *Ρ* = 0.084) and age (*t* = 1.858, *Ρ* = 0.064) between the two groups (Table 2). All of the six SNPs were in Hardy-Weinberg Equilibrium in both case and control groups.

**Table 2:** The characteristics of participants

Group	Case (***n*** = 233)	Control (***n*** = 342)	***χ***^***2***^***/t***	***Ρ***
**Gender [n(%)]**				
**Male**	110 (47.21)	160 (46.78)	0.041	0.084
**Female**	123 (52.79)	182 (53.22)		
**Age(Χ ± S)**	32.94 ± 10.77	31.30 ± 10.13	1.858	0.064

### Genotypic and allelic distributions of *EAAT1* and *EAAT2* genes in patients and controls

Difference in the allelic distribution of rs2731880 in *EAAT1* gene was observed (χ2 = 4.205, *Ρ* = 0.040), while there was no significance after Bonferroni correction (*P* < 0.008 correcting for 6 tests). The genotypic and allelic distributions of rs12294045 in *EAAT2* gene were significantly different between cases and controls (χ2 = 8.054, *Ρ* = 0.018; χ2 = 8.144, *Ρ* = 0.004, respectively). When correcting for multiple testing, the difference of allele distribution remained significant and the effect of difference of genotypic distribution was only at a trend level of significance. No association was found between schizophrenia and other four SNPs tested (*EAAT1*:rs2269272; *EAAT2*: rs3088168, rs10836387, rs12360706).

The comparison of allelic and genotypic distributions of six SNPs in *EAAT1* and *EAAT2* genes between patients with schizophrenia and controls are presented in Tables 3 and 4 respectively.

**Table 3:** Comparison of allelic distributions of six SNPs in *EAAT1* and *EAAT2* genes between patients and controls

Gene	SNP	Allele	Case	Control	***χ***^***2***^	***P***
**EAAT1**	**rs2269272**	C	321 (0.689)	474 (0.693)	0.022	0.881
		T	145 (0.311)	210 (0.307)		
	**rs2731880**	C	274 (0.588)	443 (0.648)	4.205	**0.040**
		T	192 (0.412)	241 (0.352)		
**EAAT2**	**rs3088168**	C	189 (0.406)	313 (0.458)	3.050	0.081
		T	277 (0.594)	371 (0.542)		
	**rs10836387**	G	160 (0.343)	252 (0.368)	0.758	0.384
		A	306 (0.657)	432 (0.632)		
	**rs12294045**	C	330 (0.708)	535 (0.782)	8.144	0.004*
		T	136 (0.292)	149 (0.218)		
	**rs12360706**	G	376 (0.807)	559 (0.876)	0.197	0.657
		A	90 (0.193)	125 (0.124)		

Notes: Bolded indicates statistically significant (*p* < 0.05)

*indicates the difference was still significant after Bonferroni correction

**Table 4:** Comparison of genotypic distributions of six SNPs in *EAAT1* and *EAAT2* genes between patients and controls

Gene	SNP	Genotype	Case	Control	***χ***^***2***^	***P***
**EAAT1**	**rs2269272**	CC	113 (0.485)	164 (0.480)		
		TT	25 (0.107)	32 (0.094)	0.394	0.821
		CT	95 (0.408)	146 (0.426)		
	**rs2731880**	CC	82 (0.352)	145 (0.424)		
		TT	41 (0.176)	44 (0.129)	4.106	0.128
		CT	110 (0.472)	153 (0.447)		
**EAAT2**	**rs3088168**	CC	37 (0.159)	67 (0.196)		
		TT	81 (0.348)	96 (0.281)	3.313	0.191
		CT	115 (0.493)	179 (0.523)		
	**rs10836387**	GG	32 (0.137)	41 (0.120)		
		AA	105 (0.451)	131 (0.383)	4.043	0.132
		GA	96 (0.412)	170 (0.497)		
	**rs12294045**	CC	117 (0.502)	211 (0.617)		
		TT	20 (0.086)	18 (0.053)	8.054	**0.018**
		CT	96 (0.412)	113 (0.330)		
	**rs12360706**	GG	150 (0.644)	227 (0.664)		
		AA	7 (0.030)	10 (0.029)	0.249	0.883
		GA	76 (0.326)	105 (0.307)		

Notes: Bolded indicates statistically significant (*p* < 0.05)

### Linkage disequilibrium and haplotypes

**Fig. 1:**
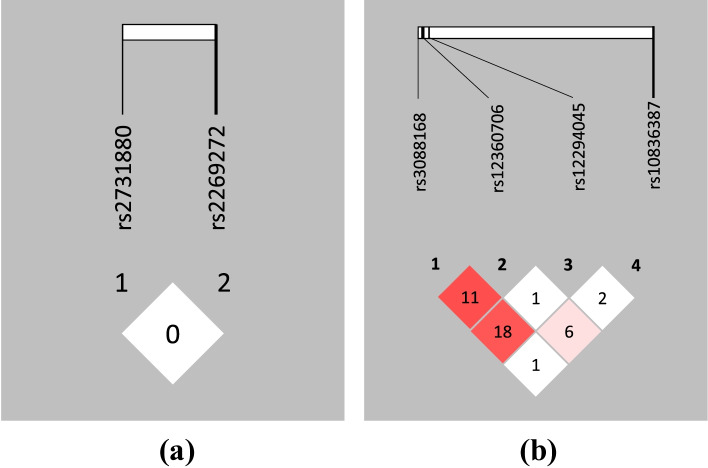
Linkage disequilibrium analysis of the selected SNPs in patients. **a** is for EAAT1 gene and **b** is for EAAT2 gene. (Note: Numbers in the LD blocks are the values of *R*^2^ which are shown as figure of percentage. For example, 11 represents *R*^2^ = 0.11)

**Fig. 2:**
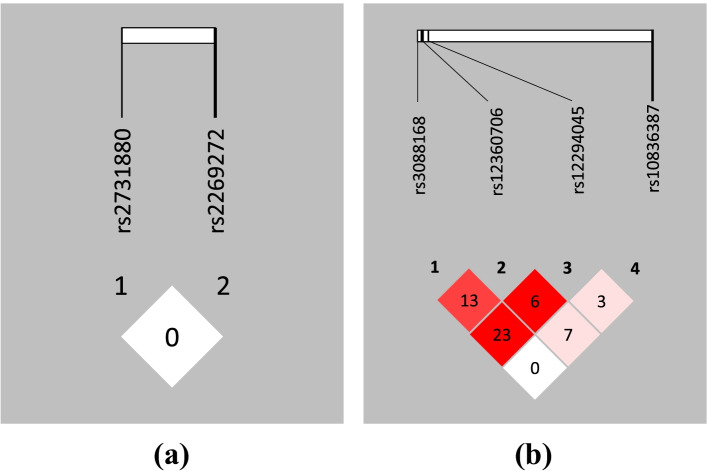
Linkage disequilibrium analysis of the selected SNPs in controls. Figure 1a is for EAAT1 gene and **b** is for EAAT2 gene. (Note: Numbers in the LD blocks are the values of *R*^2^ which are shown as figure of percentage. For example, 13 represents *R*^2^ = 0.13)

In both patients and controls pair-wise linkage disequilibrium (LD) between the investigated EAAT1 rs2269272 and rs2731880, and EAAT2 rs3088168, rs10836387, rs12294045 and rs12360706 SNPs was determined. LD blocks of EAAT1and EAAT2 genes SNPs and values of the correlation coefficient *R*^2^ for both groups are presented in Figs. 1 and 2.

No haplotype was found by haplotype analysis using the current studied loci of either *EAAT1* or *EAAT2* gene.

### Comparison of clinical features and symptoms severity with different genotypes of rs12294045

According to the above results, we found that the polymorphism of rs12294045 in *EAAT2 gene* may be associated with schizophrenia. As a result, we further examined the differences among three genotypes of rs12294045 in clinical data (gender, age of onset, onset form, total disease duration, family history of psychosis, interpersonal relationship, premorbid characteristics, current marital status, occupational status) and PANSS scores (PANSS positive scale score, PANSS negative scale score, PANSS psychopathology scale score, PANSS total score) of patients with schizophrenia. The results yielded nominally significant *P* value in family history *(P* = 0.046). Compared with patients without family history, patients with family history of psychosis had lower proportion of CC homozygous genotype and higher proportion of CT heterozygous genotype. However, *P* value did not withstand Bonferroni’s correction for multiple testing (*P* < 0.0038 correcting for 13 tests). There was no significant difference in other clinical data and PANSS scores among the three genotypes of rs12294045 (Table 5).

**Table 5:** Comparison of clinical features and symptoms severity in patients with different genotypes of rs12294045

Items	Classification	CC(117)	TT(20)	CT(96)	***χ***^***2***^/***F***	***P***
**Gender**	Male(110) Female(123)	53 (0.48) 64 (0.52)	9 (0.08) 11 (0.09)	48 (0.44) 48 (0.39)	0.510	0.775
**Age of onset** **(years, Χ ± S)**		24.06 ± 7.77	26.70 ± 7.14	23.99 ± 6.90	1.205	0.302
**Onset form**	Acute(22) Subacute(28) Chronic(183)	10 (0.46) 14 (0.50) 93 (0.51)	4 (0.18) 0 (0) 16 (0.09)	8 (0.36) 14 (0.50) 74 (0.40)	5.560	0.235
**Duration** **(months, Χ ± S)**		97.94 ± 96.04	109.00 ± 105.4	99.56 ± 96.45	0.111	0.895
**Family history**	Positive(162) Negative(71)	73 (0.45) 44 (0.62)	14 (0.09) 6 (0.08)	75 (0.46) 21 (0.30)	6.162	**0.046**
**Interpersonal relationship**	Good(15) General(123) Poor(95)	8 (0.53) 67 (0.54) 42 (0.44)	2 (0.13) 11 (0.09) 7 (0.08)	5 (0.34) 45 (0.37) 46 (0.48)	3.794	0.435
**Premorbid character**	Extro(37) Neutral(14) Intro(182)	20 (0.54) 7 (0.50) 90 (0.49)	2 (0.05) 1 (0.07) 17 (0.09)	15 (0.41) 6 (0.43) 75 (0.41)	0.734	0.947
**Marital status**	Unmarried(116) Married(75) Living apart(10)Divorced(28) Loss of spouse(4)	59 (0.51) 39 (0.52) 5 (0.50) 14 (0.50) 0 (0)	8 (0.07) 6 (0.08) 3 (0.30) 2 (0.07) 1 (0.25)	49 (0.42) 30 (0.40) 2 (0.20) 12 (0.43) 3 (0.75)	11.423	0.179
**State of occupation**	Full-time(64) Part-time(38) Jobless*(93) Unemployment*(9) Retired(28) Other works(1)	32 (0.50) 19 (0.50) 43 (0.46) 7 (0.78) 15 (0.54) 1 (1)	5 (0.08) 3 (0.08) 9 (0.10) 0 (0) 3 (0.10) 0 (0)	27 (0.42) 16 (0.42) 41 (0.44) 2 (0.22) 10 (0.36) 0 (0)	5.027	0.889
**PANSS(Χ ± S)**	Positive score	27.74 ± 4.17	28.05 ± 4.05	28.52 ± 5.40	*1.299*	0.541
**PANSS(Χ ± S)**	Negative score	23.72 ± 5.45	24.10 ± 4.52	23.80 ± 5.11	0.046	0.955
**PANSS(Χ ± S)**	psychopathology	47.74 ± 7.02	49.80 ± 7.75	48.44 ± 7.12	0.798	0.452
**PANSS(Χ ± S)**	Total score	99.20 ± 11.46	101.95 ± 10.43	100.76 ± 11.35	0.801	0.450

Notes: Bolded indicates statistically significant (*p* < 0.05); *Italics* indicates Kruskal-Wallis test for non-parametric test, and the statistic is χ^2^. (Χ ± S) means (Mean ± Standard Deviation)

*“Jobless” represents patients whose job were lost because of disease. “Unemployment” represents patients who had never worked

### Comparison of cognitive functions with different genotypes of rs12294045

One way ANOVA was performed to find out whether different genotypes of rs12294045 were related to cognitive functions of patients. Differences in Letter-Number Span Test (LNS) and Hopkins Verbal Learning Test-Revised (HVLT-R) were observed (LNS: *Ρ* = 0.016; HVLT-R: *Ρ* = 0.042). LSD analysis showed that patients with CT genotypes had significantly lower scores than those with CC and TT genotypes in terms of LNS representing working operational memory (*Ρ*1 = 0.042, *Ρ*2 = 0.011). In addition, patients with CT genotypes had significantly lower HVLT-R scores than those with TT genotypes (*Ρ*2 = 0.020) and lower scores than those with CC genotypes (*Ρ*1 = 0.098). These results suggested that the heterozygous status of rs12294045 (CT) might be considered as a risk factor for cognitive function of schizophrenia. However, after Bonferroni correction *(P* < 0.005 correcting for 10 tests), these differences were no longer significant. No significant difference was found in other subtests of MCCB (Table 6).

**Table 6:** Comparison of cognitive test scores in patients with different genotypes of rs12294045

Subtests		rs12294045		***F***	***Ρ***	***Ρ*** 1	***Ρ*** 2	***Ρ*** 3
	**CT(*n*** **= 96)**	**CC(*n*** **= 116)**	**TT(*n*** **= 20)**					
**TMT-A**	45.23 ± 7.86	46.28 ± 11.97	47.95 ± 7.54	0.704	0.495	0.442	0.274	0.500
**Symbol Coding**	42.00 ± 10.56	40.98 ± 10.10	44.60 ± 11.07	1.113	0.330	0.473	0.308	0.149
**Category Fluency**	47.75 ± 11.32	47.35 ± 11.25	48.55 ± 10.88	0.105	0.901	0.805	0.772	0.664
**CPT-IP**	41.84 ± 11.11	42.05 ± 10.75	45.25 ± 13.08	0.823	0.441	0.914	0.213	0.228
**LNS**	43.76 ± 10.97	46.96 ± 11.32	50.75 ± 9.89	4.184	**0.016**	**0.042**	**0.011**	0.149
**WMS-III**	44.34 ± 12.03	44.68 ± 12.93	47.10 ± 12.63	0.413	0.662	0.914	0.373	0.399
**HVLT-R**	42.22 ± 12.18	44.94 ± 10.88	48.80 ± 11.08	3.222	**0.042**	0.098	**0.020**	0.154
**BVMT-R**	43.89 ± 11.79	43.16 ± 11.16	44.30 ± 13.07	0.192	0.826	0.598	0.884	0.655
**NAB-MAZES**	43.02 ± 8.99	42.94 ± 11.05	42.65 ± 10.70	0.013	0.987	0.914	0.883	0.930
**MSCEIT**	47.03 ± 12.30	48.09 ± 11.91	51.75 ± 14.55	1.222	0.297	0.568	0.120	0.209

Note: Bolded indicates statistically significant (*p* < 0.05)

*Ρ* 1 represents the comparison between CT genotype patients and CC genotype patients; *Ρ* 2 represents the comparison between CT genotype patients and TT genotype patients; *Ρ* 3 represents the comparison between CC genotype patients and TT genotype patients.(*TMT-A* Trail Making Test A, *CPT-IP* Continuous Performance Test-Identical Pairs, *LNS* Letter-Number Span Test, *WMS-III* Wechsler Memory Scale-Third Edition, *HVLT-R* Hopkins Verbal Learning Test-Revised, *BVMT-R* Brief Visuospatial Memory Test-Revised, *NAB* Neuropsychological Assessment Battery, *MSCEIT* Mayer-Salovey-Caruso Emotional Intelligence Test).

## Discussion

There are two objectives of this study. Firstly, the relationship between *EAAT1* and *EAAT2* genes and schizophrenia was explored. We found no significant association between *EAAT1* gene polymorphism and schizophrenia. For *EAAT2* gene, the polymorphism of rs12294045 may be associated with schizophrenia in Chinese Han population. Secondly, we investigated the relationship between the polymorphism of rs12294045 and clinical characteristics, symptoms severity and cognitive function in patients with schizophrenia. The results showed that rs12294045 locus may be associated with family history, working operational memory and verbal learning, in which the CT genotype was a risk factor. However, no significance remained after multiple testing.

In this study, only a trend was showed that the allele distribution of rs2731880 in *EAAT1* gene may be associated with schizophrenia. Previous studies reported the relation between EAAT1 and schizophrenia. Spangaro M et al. found that rs2731880 in European population may be related to schizophrenia [25]. In addition, the levels of EAAT1 mRNA expression in the thalamus of subjects with schizophrenia was found significantly higher than healthy subjects [34]. Animal model studies [35] showed that compared with wild-type mice in the same nest, *EAAT1* knockout mice have negative social behaviors, such as lack of pleasure, social withdrawal and self-neglect. Our inconsistent results may be because of the small sample size or the genetic differences among different races.

Our study found an evidence about the association between rs12294045 in EAAT2 gene and schizophrenia. There were few previous studies on rs12294045 and schizophrenia. Appenzeller S et al. [36] explored the polymorphism of rs12294045 in Parkinson’s disease and no association was found. For the relationship between EAAT2 gene and schizophrenia, recent studies have shown different results. A study on EAAT2 mRNA have found that region-specific increases in cortical EAAT2 mRNA were involved in schizophrenia pathophysiology [18]. A case-control study in Japanese population [24] reported the *EAAT2* gene polymorphism was associated with schizophrenia, and concluded that at least one of the susceptibility locus for schizophrenia may reside in internal or nearby *EAAT2*. In contrast, no association was observed between the polymorphism − 181 A/C (rs4354668) in SLC1A2 (EAAT2) and onset of schizophrenia and its psychopathology in Polish population [37].

With regard to the relationship between *EAAT2* gene and cognitive function in schizophrenia patients, our findings were fairly weak. Previous studies have shown that working memory represents the core content of changes in schizophrenia, it has been indicated that working memory can predict the severity of cognitive impairment and play a key role in the performance of other cognitive tasks in patients with schizophrenia [38, 39]. The results of prospective studies suggested that patients carrying the G allele of *EAAT2* SNP rs4354668 had significantly reduced gray matter volume and impaired working memory function [26]. Spangaro M et al. [40] found that genotypes associated with low expression of EAAT2 were significantly associated with cognitive dysfunction such as executive function and working memory function. In agreement with the above findings, recent studies also suggested the genetic variation in EAAT2 may be involved in impaired working memory and executive function of patients with schizophrenia [41, 42]. Further study on the function of rs12294045 locus to explore how its polymorphism influences the expression of EAAT2 mRNA is needed, which could help us better understand the relationship between this locus and cognitive dysfunction in patients with schizophrenia.

Our findings are subject to a number of limitations. First, the sample size of this study was small which led to a relatively low genetic power. Second, our patients were identified through the inpatient department of hospital; we didn’t include schizophrenia patients in the outpatient clinics. Our findings may not generalize to patients who don’t seek treatment or those who don’t need hospitalization. Third, only one method (MCCB) was used to test the cognitive function of patients, so the patients’ cognitive dysfunction may not be fully explored. Fourth, we could not completely eliminate other possible factors, such as the previous medical treatment. Last but not least, *EAAT1* and *EAAT2* genes loci were still not identified by genome-wide association analysis in the most recent schizophrenia study [43]. Schizophrenia is a complex disease with unknown etiology and many factors could contribute to the pathogenesis of the disease. Therefore, genome-wide association study (GWAS) of schizophrenia based on larger sample size and interaction study between genes and other genetic and environmental factors are further needed.

## Conclusions

In our study, the polymorphism of rs12294045 locus in *EAAT2* gene may be associated with schizophrenia. CT genotype may be one of the risk factors for family history, the dysfunction of working operational memory and verbal learning in patients.

## Data Availability

Researchers interested in the study may contact corresponding author to obtain relevant data via email: liulf521@163.com.

## Abbreviations

CNS: Central Nervous System
SNPs: Single nucleotide polymorphisms
EAAT: Excitatory Amino Acid Transporter
NMDA: N-methyl-D-aspartate
MAF: Minor Allele Frequency
PCR: Polymerase Chain Reaction
DSM-IV: Fourth Edition of the Diagnostic and Statistical Manual for Mental Disorders
PANSS: Positive and Negative Syndrome Scale
MCCB: Matrics Consensus Cognitive Battery
HWE: Hard-Weinberg Equilibrium
LD: linkage disequilibrium
BVMT-R: Brief Visuospatial Memory Test-Revised
CPT-IP: Continuous Performance Test-Identical Pairs
HVLT-R: Hopkins Verbal Learning Test-Revised
LNS: Letter-Number Span Test
MSCEIT: Mayer-Salovey-Caruso Emotional Intelligence Test
NAB: Neuropsychological Assessment Battery
TMT-A: Trail Making Test A
WMS-III: Wechsler Memory Scale-Third Edition
